# Long-Term Observation of Triplex Surgery for Cataract after Phakic 6H Implantation for Super High Myopia

**DOI:** 10.1155/2016/9569868

**Published:** 2016-04-14

**Authors:** Xin Liu, Fan Fan, Xiaoying Wang, Yi Lu, Tianyu Zheng, Peng Zhou, Xingtao Zhou, Yi Luo

**Affiliations:** ^1^Department of Ophthalmology, Eye and ENT Hospital, Fudan University, Shanghai 200032, China; ^2^Department of Ophthalmology, ParkwayHealth Hong Qiao Medical Center, Shanghai, China

## Abstract

*Purpose*. To analyze the safety, effectiveness, and stability of triplex surgery for phakic 6H anterior chamber phakic intraocular lens explantation and phacoemulsification with in-the-bag IOL implantation for super high myopia in long-term observations.* Methods*. This retrospective case series evaluated 16 eyes of 10 patients who underwent triplex surgery. Best corrected visual acuity (BCVA), endothelial cell density (ECD), and associated adverse events were evaluated.* Results*. The mean follow-up time after the triplex surgery was 46 ± 14 months. The mean logMAR BCVA was significantly improved after triplex surgery (*P* = 0.047). One eye developed endophthalmitis five days postoperatively and underwent pars plana vitrectomy (PPV). Five eyes with preoperative severe endothelial cell loss developed corneal decompensation and underwent keratoplasty at a mean time of 9.4 ± 2.6 months after the triplex surgery. One eye had graft failure and underwent a second keratoplasty. The eye developed rhegmatogenous retinal detachment and underwent PPV with silicone oil 18 months later. ECD before the triplex surgery was not significantly different compared with that at last follow-up (*P* = 0.495) apart from these five eyes. Three eyes (18.8%) developed posterior capsule opacification.* Conclusions*. Triplex surgery was safe and effective for phakic 6H related complicated cataracts. Early extraction before severe ECD loss is recommended.

## 1. Introduction

Anterior chamber angle-supported phakic intraocular lenses (AC pIOLs) were once widely accepted as an effective option for the correction of high myopia [1]. They offer accurate and stable refraction, preserve the shape of the cornea, preserve accommodation of the crystalline lens, and are potentially reversible compared to laser keratorefractive or refractive lens exchange procedures [2]. The early generation PMMA AC pIOL phakic 6H has a particularly wide optic diameter (6.0 mm, 5.5 mm in powers greater than −20.0 D) and needed a large incision for implantation [3]. Its visual outcomes are encouraging. The best corrected visual acuity (BCVA), refraction, and contrast sensitivity are all improved in the short-term reports [4–6]. It was used globally since 1990s and was popular in China since 2002. However, this model was phased from the market because its use was associated with progressive ovalization of the pupil, glare, and endothelial cell loss in the long run [2, 7]. In China, phakic 6H was withdrawn in early 2012 and patients with previous implantation suffered prolonged endothelial cell loss and pupil ovalization [8]. Furthermore, with aging and progression of pathological high myopia, cataract formation, accompanied by severe problems involving the iris and cornea, develops and calls for AC pIOL explantation and vision restoration [9].

Surgical outcomes of patients with new-onset AC pIOL-associated cataracts (called bilensectomy surgery, and mostly for ZB5M and ZSAL-4 model) are usually successful after less than 1 year of mean follow-up time [10, 11]. In the present study, we applied triplex surgery of phakic 6H AC pIOL explantation and phacoemulsification with in-the-bag intraocular lens (IOL) implantation in order to solve phakic 6H AC pIOL-related complications. We provide the longest follow-up term after explantation of AC pIOLs and evaluate its safety, effectiveness, and stability, which might alert ophthalmologists to closer postoperative surveillance and help surgeons to determine the optimal surgical time.

## 2. Patients and Methods

### 2.1. Patients

We retrospectively reviewed 16 eyes of 10 patients who were previously implanted with phakic 6H AC pIOL (Ophthalmic Innovations International, Ontario, CA) for the correction of super high myopia and developed lens opacity undergoing triplex cataract surgery from July 1, 2008, to June 30, 2012, at Eye & ENT Hospital of Fudan University, Shanghai, China. We also included one patient (Case 2) who underwent phakic 6H AC pIOL implantation at a different hospital and developed cataract. This patient was transferred to our cataract clinic for triplex surgery during the above period. Written informed consent was obtained from all patients. This study was carried out with the approval of the Institutional Review Board of Eye and ENT Hospital of Fudan University and in accordance with the Declaration of Helsinki.

### 2.2. Preoperative Evaluation

The following examinations were performed preoperatively: best corrected visual acuity (BCVA), slit-lamp biomicroscopy, dilated fundus examination, intraocular pressure (IOP), axial length (AL), and endothelial specular microscopy. Cataract type and morphology were evaluated using the Lens Opacities Classification System III (LOCS III) [12]. AL and anterior segment size were measured using interferometry (IOL Master, Carl Zeiss Meditec AG) or A-scan ultrasonography without any correction for the pIOL* in situ*. The SRK/T or Holladay 1 formula was used for posterior chamber IOL calculations and the targeted refraction was emmetropic to low myopic as directed by the patients.

Triplex surgery was deemed necessary when BCVA decreased by at least 2 lines from the level achieved after phakic 6H AC pIOL implantation and was related to cataract or when the endothelial cell count decreased markedly to 1500 cells/mm^2^ in eyes with mild lens sclerosis (C1/N1/P1 by LOCS III). When ECD reached 1500 cells/mm^2^, triplex surgery was performed in an effort to halt the progression of ECD loss. If the cornea retains transparency after the triplex surgery, keratoplasty is not necessary. However, when corneal decompensation was inevitable, the triplex surgery was performed followed by keratoplasty.

### 2.3. Surgical Techniques

All the triplex surgeries were performed by 1 of 2 surgeons (Yi Luo and Yi Lu). On the day of surgery, the patients were given phenylephrine hydrochloride 0.5% and tropicamide 0.5% eye drops to dilate the pupil. After retrobulbar anesthesia, a 3.2 mm superior or temporal scleral tunnel incision and a 1.0 mm side-port incision were made. For patients implanted with phakic 6H AC pIOLs who suffered from peripheral anterior synechiae (PAS), goniosynechialysis was performed. The scleral incision was then enlarged to 7 mm. DisCoVisc (Alcon Laboratories, USA) was injected into the anterior chamber to protect the endothelium, followed by dislocation of the proximal haptics of the phakic 6H AC pIOL to the scleral wound by gentle rotation using a Bechert lens manipulator. The AC pIOL was then grasped with forceps and extracted from the anterior chamber. The incision was partially closed with two 10-0 nylon sutures. After the AC pIOL was explanted, standard bimanual phacoemulsification and placement of a posterior chamber IOL in the lens capsule were performed. One of the following posterior chamber IOLs was implanted: AcrySof MA60MA (Alcon Laboratories, USA), Rayner 620H (Buromed, England), or HumanOptics MC X11 ASP (Germany) IOL. Tobramycin-dexamethasone drops were administered to all patients for 2 weeks and pranoprofen ophthalmic solution was used 3 times daily for 1 month.

### 2.4. Follow-Up

Changes in endothelial cell density (ECD), IOP, BCVA, and intraoperative and postoperative adverse events were measured. Patients were examined 1 day, 1 week, and 1, 3, and 6 months postoperatively and thereafter every 6 months for at least 2 years.

### 2.5. Statistical Analysis

All statistical analyses were performed using SPSS 16.0 (SPSS Inc., Chicago, IL, USA). Visual acuity was determined using Snellen charts, and logMAR values were used for the calculations. Categorical variables were expressed as numbers and percentages. Continuous variables were expressed as the mean ± standard deviation. Categorical variables were compared between groups using Fisher's test, while numerical variables were compared using Student's* t*-test or Wilcoxon rank-sum test for independent samples and paired Student's* t*-test for paired samples. The level of significance was 0.05.

## 3. Results

### 3.1. Baseline Data

Patients had a mean age of 38 ± 6 years (range: 27 to 37 years) at the time of triplex surgery. The mean follow-up time after the triplex surgery was 46 ± 14 months (range: 24 to 67 months). Baseline clinical data before and after phakic 6H AC pIOL implantation in eyes that developed cataract and underwent the triplex surgery are presented in Table 1. Clinical data before and after the triplex surgery are presented in Table 2.

Nine eyes (56.3%) underwent posterior scleral reinforcement surgery before phakic 6H AC pIOL implantation. One patient (Case 2) had hyperthyroidism 4 years after phakic 6H AC pIOL implantation and developed thyroid eye disease (TED) with signs of bilateral eyelid retraction and mild lagophthalmos. One patient (Case 5) developed diabetes mellitus 3 years after phakic 6H AC pIOL implantation. Problems involving the cornea and iris were common in eyes with phakic 6H AC pIOL implantation. Four eyes (Cases 1 and 2) had mild corneal edema. Nine eyes (56.3%) suffered pupil ovalization with different degrees of PAS and iris atrophy. Two eyes (right eye of Cases 4 and 5) had mild chronic anterior chamber inflammation. Improper diameter selection led to an undersized (Case 2) pIOL. Rotation and decreased space between the AC pIOL and endothelium threatened the endothelium and caused excessive endothelial cell loss.

### 3.2. Intraoperative Data

During the triplex surgery, three eyes (Case 1 and left eye of Case 3) had severe pupil ovalization with severe PAS. The haptics of the phakic 6H AC pIOLs tightly adhered to the peripheral iris and anterior chamber angle. Separation and dislocation of the haptics of pIOLs were performed by two IOL manipulators after injection of DisCoVisc. Mild anterior chamber hemorrhage occurred in 1 eye (Case 3) because of injury to the anterior chamber angle. The haptics of AC pIOLs were found to be touching the corneal endothelium in two eyes (Case 2). Two eyes had hard nuclear cataract (N4C2P1 by LOCS III) and the power of phacoemulsification was 12.5% for 21 seconds (left eye of Case 1) and 21.0% for 30 seconds (Case 7), respectively. No vitreous loss or capsule rupture occurred in any eye.

### 3.3. Postoperative Adverse Events

One eye (Case 2L) presented with ocular pain, loss of visual acuity to hand movements, corneal edema, and anterior chamber fibroblastic reaction and was diagnosed with acute endophthalmitis 5 days after the triplex surgery. The eye immediately received a vitreous tap and intravitreal antibiotics (0.8 mg/0.1 mL Vancomycin and 2.25 mg/0.1 mL Ceftazidime) injection. No sign of recovery was observed the following day and the eye immediately underwent a pars plana vitrectomy and received the same intravitreal antibiotic injection. Inflammation was then resolved. Vitreous culture showed a* Staphylococcus auricularis* infection. Five eyes (31.3%) of three patients (Cases 1, 2, and 3L) with severe preoperative endothelial cell loss developed corneal decompensation and underwent penetrating keratoplasty (PKP) or Descemet's stripping automated endothelial keratoplasty (DSAEK) at a mean time of 9.4 ± 2.6 months (range: 6 to 13 months) after the triplex surgery. The clinical data and characteristics of these eyes are presented in Table 3. Case 2L experienced early graft rejection with corneal edema and steroid-induced IOP elevation after DSAEK. The endothelial pathology expanded to whole-layer pathology, with subepithelial scarring and stromal opacity. PKP was performed after graft failure. After 18 months, Case 2L developed rhegmatogenous retinal detachment (RRD) and underwent PPV with silicone oil. Cases 1L and 3L both experienced two late graft rejection episodes. These late episodes correspond to reduction of topical steroid use due to steroid-response glaucoma and poor compliance, respectively. Hourly doses of topical prednisolone acetate 1% while awake and antiglaucoma medications when necessary were prescribed. The late rejection episodes and elevated IOP were controlled using only medication. Graft detachment occurred in Case 1R and air reinjection was performed 1 week after DSAEK. However, peripheral partial detachment < 1/8 in the supratemporal region still existed. It did not influence the visual axis and mostly spontaneously improved during the follow-up period. The above complications were all managed adequately and all corneas maintained transparency at last follow-up. Three eyes (18.8%) of 3 patients (Cases 4L, 5L, and 6L) developed PCO and were treated with Nd:YAG laser posterior capsulotomy at a mean time of 17.0 ± 5.6 months (range: 12 to 23 months) after triplex surgery.

### 3.4. Visual Outcomes

The mean logMAR BCVA was significantly improved after phakic 6H AC pIOL implantation (*P* = 0.001) and was significantly reduced after cataract formation* (P* < 0.001). BCVA was significantly improved after triplex surgery (*P* = 0.047). The difference between BCVA at last follow-up and that 6 months after phakic 6H AC pIOL implantation was not significant (*P* = 0.075). Comparison between eyes that developed corneal decompensation and those that did not revealed worse BCVA (logMAR) at last follow-up (*P* < 0.0001). BCVA at last follow-up did not significantly differ between eyes with PCO and those without (*P *= 0.057). The mean final manifest spherical equivalent was −1.61 ± 0.95 D (range: −3.75 to −0.25 D) after the triplex surgery. Thirteen eyes (81.3%) were within ±1.0 D of the intended correction.

### 3.5. ECD Change

ECD was significantly reduced after phakic 6H implantation (*P* = 0.004) and ECD loss significantly progressed over time (*P* < 0.001). The mean percentage of ECD loss was 26.4% over the mean period of 6.6 ± 1.2 years (range: 4.0 to 8.3 years) after phakic 6H AC pIOL implantation. A subgroup analysis of eyes with severe endothelial cell loss before the triplex surgery is presented in Table 3. Apart from the five eyes that later developed corneal decompensation, ECD before the triplex surgery was not significantly different compared with ECD at the last follow-up (2144 ± 468 cells/mm^2^ versus 2103 ± 425 cells/mm^2^,* P* = 0.495). The triplex surgery effectively stabilized early ECD loss.

Comparison between eyes that developed corneal decompensation and those that did not revealed lower ECD after phakic 6H AC pIOL implantation (*P* = 0.007), before triplex surgery (*P* = 0.041) and at last follow-up (*P* < 0.0001). All eyes that developed corneal decompensation suffered pupil ovalization. All eyes with corneal edema developed corneal decompensation.

## 4. Discussion

The present study showed that triplex surgery of phakic 6H AC pIOL explantation and phacoemulsification with in-the-bag IOL implantation was an effective means for improvement of vision and refraction in agreement with previous studies [9–11]. The current study investigated the safety, effectiveness, and stability of triplex cataract surgery. To the best of our knowledge, our study provided the longest follow-up time after AC pIOL explantation for cataract and the largest case series report for the specific phakic 6H model explantation for cataract. AC pIOL-related severe ECD loss leading to corneal decompensation, corneal edema and pupil ovalization, inflammation, and corneal damage induced by complications during the perioperative period and systemic diseases are issues confronted and needed to be evaluated comprehensively when making the decision of triplex surgery. PCO was another frequent postoperative adverse event. Closer surveillance for young patients in long-term follow-up is needed.

Cataract formation is one of the major complications of pIOLs implantation [13] and is accelerated by super high myopia [14]. In the current study, the mean age of patients at the time of triplex surgery was 38.5 years and the mean axial length was 31.63 mm. There is an increased risk of cataractogenesis after AC pIOL implantation in patients older than 40 years that have an axial length longer than 29 mm [10]. In our study, the mean time between phakic 6H AC pIOL implantation and triplex surgery for cataract was 6.6 ± 1.2 years. All the cataracts types found in eyes that underwent the triplex surgery were nuclear. Alió et al. reported that the mean interval between implantation and explantation of AC pIOLs (62 with ZB5M, 1 with ZSAL-4, and 1 with phakic 6H) due to cataract formation was 10.04 ± 3.66 years [11]. In their cases, almost 100% of cataracts detected in the eyes that underwent angle-supported AC pIOL explantation surgery were nuclear [9, 11]. Phakic AC pIOL implantation promotes early changes of the nucleus because of chronically inadequate aqueous perfusion to the lens epithelium, surgical trauma, chronic subclinical inflammation, and postoperative use of topical steroids [13, 15, 16]. Cataract is also the main cause of AC pIOL explantation and was reported to represent 51.39% of the cases of explantation [9]. The increase in cataract with aging and progression of pathogenetic high myopia is a concern and suggests caution regarding long-term safety.

The triplex surgery was an effective means for visual restoration. Mean BCVA was significantly improved after triplex surgery and was not significantly different from that after phakic 6H implantation. The mean final manifest spherical equivalent was −1.61 D. Previous study also demonstrated that final BCVA was not significantly different after cataract surgery in 9 highly myopic eyes corrected by ZB5M and ZSAL-4 AC pIOL from that after AC pIOL implantation. SE was −0.42 ± 1.94 D after cataract surgery [10]. However, the BCVA and SE were significantly different after bilensectomy surgery in a larger case series [9]. Our results were comparable to these results.

Corneal endothelial cell loss is the main concern after AC pIOL implantation [7]. In the current study, eyes suffered progressive ECD loss (26.4% over 6.6 years) after phakic 6H implantation. Alió et al. found that severe endothelial cell loss leading to AC pIOL explantation was related to the use of phakic 6H pIOL in the short term (3.22 years, which was much shorter than that for ZB5M cases) [11]. The reported percentage of ECD loss after AC pIOLs implantation was 3.8% to 12% in the first year and gradually decreased by 0.5% to 1.8% per year [7]. The endothelial issues are controversial and evoke substantial debate [3]. Damage to the corneal endothelium mainly results from direct contact between AC pIOLs and the inner surface of the cornea and inflammation [7]. Removal of the pIOL based on progressive loss of endothelial cells or reaching an absolute value (i.e., 1500 cells/mm^2^), at which point the eye may have decreased ability to sustain other types of surgery, is debatable in the absence of a clear guideline. Corneal integrity depends on the absolute number of endothelial cells and their function and morphology. Apart from the five eyes that later developed corneal decompensation, the triplex surgery effectively stabilized early ECD loss. When severe ECD loss occurred, especially accompanied by other relative complications or significant corneal morphology change, primary keratoplasty with AC pIOL explantation might be a better alternative [17, 18].

Complications during the perioperative period and systemic diseases are other core issues that needed to be evaluated comprehensively when making the decision of triplex surgery. Pupillary ovalization was detected in 56.3% of cases that underwent the triplex surgery. Different degrees of PAS and iris atrophy were also common. The haptics of AC pIOLs in the sclerocorneal angle lead to mild deformation of the iridosclerocorneal architecture, resulting in iris retraction and pupil ovalization [17]. The haptic plate of the pIOL adhered to the tissue in the anterior chamber angle. These problems increased the difficulty of dissection and dislocation of the pIOL haptic and elevated the inflammatory reaction, which might cause direct toxicity to the endothelium and angle. A soft-shell technique, higher molecular weight, and viscoelastic and other procedures should be used to protect the corneal endothelium during surgery. Furthermore, considering the long life expectancy of the patients with implanted pIOLs at a younger age, development of systemic disease should not be ignored.

Several reasons, categorized as either “patient-related” or “graft-related” factors, contributed to prolonged low vision of the five eyes that developed corneal decompensation. The most common patient-related causes were fundus pathology caused by super high myopic degeneration and uncorrected refractive error after keratoplasty. Incomplete visual rehabilitation was also attributed to problems related to phakic 6H, such as severe pupil ovalization and peripheral anterior synechiae. Various perioperative complications required multiple surgeries. Case 2L was the extreme case. Multiple surgical traumas led to repeated inflammation reaction and, to some extent, a change in ocular structure and function. Graft-related factors included complications after corneal intervention of DSAEK/PKP, donor factors, and surgical experience. Both early and late graft rejections associated with steroid-induced IOP elevation and poor compliance of topical corticosteroid use resulted in continuous ECD loss and one graft failure. Steroid-induced IOP elevation and glaucoma medications are risk factors for higher graft rejection and failure rates with higher ECD loss after DSAEK and PKP [19–21]. In developing countries, where there is a perpetual shortage of donor corneal tissue, corneas are often donated by older people with systemic chronic diseases. The waiting period for corneal transplantation is long. Earlier surgery, within a year of disease onset, may produce superior visual outcomes both in EK and in PKP by limiting the duration of stromal edema and reducing fibrosis [22–24]. Graft-host interface irregularity also contributed to incomplete visual rehabilitation after EK [23]. The three eyes that underwent DSAEK were among the first 50 cases of DSAEK in our hospital. The learning curve for the DSAEK procedure may influence the rate of ECD loss and incidence of graft detachment [25]. Factors associated with higher postoperative ECD loss include secondary donor reattachment procedure, episodes of graft rejection, medically treated glaucoma, and older donor age [19, 26, 27]. Patient- and graft-related factors and multiple comorbidities resulted in prolonged low vision in these five eyes warranting further intensive follow-up.

The development of PCO is a multifactorial process affected by patient-related, IOL-related, and surgery-related problems [28]. In our study, 18.8% of eyes developed PCO, which was comparable to other studies [29, 30]. The final BCVA did not significantly differ between eyes with PCO and those without. High myopia is pathological and is associated with an increase in certain growth factors in the aqueous humor, which strongly influence the development of PCO [31]. Other patient-related factors include a history of chronic inflammation caused by AC pIOL (Case 4) or related to diabetes mellitus (Case 5). Hydrophilic acrylic material offers the advantage of good uveal biocompatibility [32, 33] and is therefore suitable for patients with high myopia who undergo the triplex surgery that induces a higher inflammatory response than routine cataract surgery. PCO incidence is primarily influenced by IOL design [28, 34]. The PCO score was significantly lower with a sharp optic edge and a capsular bend formation compared to round edged IOLs [34]. However, the incomplete sharp edge at the broad optic-haptic junctions represents an Achilles heel and allows migration of lens epithelial cells [35] and is therefore associated with a poorer PCO outcome. Thus, in eyes with extreme high myopia that underwent the triplex surgery, an enhanced 360-degree sharp-edged design posterior chamber IOL implantation [35] and manual polishing of the capsule are recommended and extensive follow-up for the detection of PCO is needed.

Our study had some limitations. It is a retrospective study, associated with possible selection bias. However, it is difficult to conduct a prospective study because phakic 6H AC pIOLs have been phased out of the market. Once signs of corneal decompensation occurred, the condition was extremely complicated and changeable. The patient might require multiple surgeries and might have poor prognosis even after great effort, as in Case 2. Therefore, before triplex surgery, a comprehensive and specialist evaluation of risks associated with AC pIOL involving the iris and cornea, possible complications during the perioperative period, ocular comorbidities, and systemic diseases is of vital significance. Early extraction of phakic 6H AC pIOL before severe ECD loss is the best option. In case of severe ECD loss, especially accompanied by other related complications or significant corneal morphological changes, primary keratoplasty combined with AC pIOL explantation or triplex surgery might be a good alternative [17, 18, 36]. Further investigations comparing the two procedures are needed to determine the indications and optimal surgical time. Another limitation of our study is that the DSAEKs were among the initial 50 cases performed in our hospital. However, in real world, such experience is especially useful due to the limitations associated with keratoplasty in China [37]. Such a retrospective study enables anticipation of the most complicated problems of these cases to determine the optimal surgical time.

In conclusion, the perspective and information gained from the present study provide some basis for optimism in the management of complicated problems related to phakic 6H AC pIOLs, including cataracts. Corneal decompensation should be observed closely in eyes with progressive ECD loss presenting with pupil ovalization, corneal edema, or corneal inflammation. This is especially the case for eyes accompanying other related ocular or systemic abnormalities. The triplex surgery or simple explantation of AC pIOL should be performed early. A 360° sharp-edged design posterior chamber IOL implantation and long-term follow-up for PCO development are recommended. Triplex surgery is safe and effective for phakic 6H complicated cataract and our experience provided comprehensive evaluation of issues confronted.

## Figures and Tables

**Table 1:** Baseline clinical data before and after phakic 6H AC pIOL implantation in eyes that developed cataract and underwent the triplex surgery.

Case/eye	Sex/age (y)	AL (mm)	ACD (mm)	CCT (*µ*m)	WTW (mm)	SE		Phakic 6H AC pIOL		ECD (cells/mm^2^)		BCVA (logMAR)		Time between PSR surgery and phakic 6H implantation (y)	Time between phakic 6H implantation and triplex surgery (y)
						Pre	Post	Power	Optic diameter (mm)	Pre	Post (6 m)	Pre	Post		
1/R	F/42	31.94	3.62	501	11.3	−21.50	−2.00	−19.50	12.5	2852	2530	0.52	0.30	3.0	6.5
1/L		31.64	3.34	497	11.3	−21.50	−1.50	−19.50	12.5	2709	2451	0.52	0.30	3.0	6.5
2/R	F/35	31.04	3.52	510	11.9	−15.00	−0.50	−14.00	12.5	2403	2103	0.10	0.10	None	6.0
2/L		30.67	3.38	492	11.8	−14.00	−1.25	−13.00	12.5	2050	2100	0.22	0.22	None	6.0
3/R	F/38	29.09	3.70	519	12.0	−15.50	+0.50	−15.50	13.0	2698	2579	0.15	0.00	5.0	7.0
3/L		31.13	3.67	507	11.7	−20.50	−0.75	−19.50	12.5	2543	2541	0.40	0.22	5.0	7.0
4/R	F/47	31.09	3.58	507	12.1	−15.50	+0.50	−15.00	13.0	2954	2705	0.05	0.00	1.5	7.0
4/L		29.75	3.74	513	12.1	−13.75	−1.00	−13.50	13.0	3010	2828	0.00	0.00	1.5	7.8
5/L	F/45	33.75	3.62	558	12.7	−18.50	−2.25	−17.50	13.5	2922	2749	0.15	0.00	None	8.3
6/R	F/39	32.51	3.66	554	11.6	−25.00	0.00	−23.50	12.5	2322	2545	0.52	0.22	7.5	8.3
6/L		32.39	3.89	546	11.6	−27.00	−0.75	−23.50	12.5	2900	2708	0.52	0.22	7.5	6.3
7/R	M/44	33.72	3.89	527	11.8	−22.00	−0.25	−19.00	12.5	2373	2384	1.30	0.70	None	4.0
8/R	F/31	32.59	3.53	589	11.5	−22.75	−1.00	−21.00	12.5	2734	2699	0.40	0.30	None	5.5
8/L		32.41	3.54	574	11.4	−22.00	−0.75	−20.50	12.5	3195	3058	0.40	0.30	None	5.8
9/L	F/27	31.58	3.18	537	11.4	−26.00	−3.00	−22.00	12.5	2769	2681	0.70	0.70	None	5.0
10/L	F/37	30.73	3.62	540	11.6	−19.00	0.00	−18.00	12.5	2730	2486	0.82	0.40	6.5	8.3
Mean (SD)	38.5 (6.3)	31.63 (1.28)	3.59 (0.19)	529 (29)	11.7 (0.4)	−19.97 (4.27)	−0.89 (0.95)	−18.41 (3.40)	12.7 (0.3)	2698 (296)	2572 (245)	0.42 (0.33)	0.25 (0.22)	4.5 (2.4)	6.6 (1.2)

AL: axial length; ACD: anterior chamber depth (including corneal thickness); CCT: central corneal thickness; WTW: white to white; SE: spherical equivalent; Pre: before phakic 6H AC pIOL implantation; Post: after phakic 6H AC pIOL implantation; ECD: endothelial cell density; BCVA: best corrected visual acuity; SE: spherical equivalent; PSR: posterior scleral reinforcement surgery; m: month(s).

**Table 2:** Clinical data before and after triplex surgery.

Case/eye	Follow-up period (m)	Pupil diameter (mm)	LOCS III classification	Phacoemulsification		BCVA (logMAR)			SE after the triplex surgery	ECD (cells/mm^2^)			IOL	
				Power	Time	Pre	Post	At last follow-up		Pre	Post (1 m)	At last follow-up	Power	Model
1/R	54	5.0*∗*3.0	N3C1P1	12.0	18	0.40	0.40	1.00	−2.25	1889	1750	681	4.0	Rayner
1/L	54	5.0*∗*3.0	N4C2P1	12.5	21	0.60	0.60	1.00	−0.75	1495	1153	572	3.0	Rayner
2/R	63	4.0*∗*2.0	N3C1P1	11.3	10	0.60	1.30	1.00	−2.25	1832	1765	1015	6.0	HumanOpt
2/L	57	6.0*∗*2.0	N1C1P1	1.0	1	1.00	1.30	3.00	−3.75	1676	1298	1224	4.0	HumanOpt
3/R	36	3.0*∗*3.0	N3C1P1	9.0	13	0.52	0.10	0.10	−1.50	2122	2072	1987	5.0	Rayner
3/L	36	8.0*∗*3.0	N2C1P1	2.0	2	0.70	1.00	1.00	−0.75	1314	1215	980	2.0	Rayner
4/R	36	3.0*∗*3.0	N3C1P1	10.0	17	0.40	0.00	0.00	−1.75	2077	2105	2011	4.0	HumanOpt
4/L	26	3.0*∗*3.0	N3C2P1	12.5	21	0.52	0.00	0.00	−2.75	2228	2205	2033	6.0	HumanOpt
5/L	38	3.0*∗*3.0	N3C1P1	9.9	15	0.30	0.00	0.00	−2.50	1422	1399	1348	−2.0	HumanOpt
6/R	24	6.0*∗*2.5	N3C1P1	16.6	12	0.52	0.22	0.15	−0.50	1869	1843	1889	−1.0	HumanOpt
6/L	51	3.0*∗*3.0	N3C1P1	12.5	13	0.52	0.22	0.22	−1.75	2028	2023	1988	−2.0	HumanOpt
7/R	67	6.0*∗*3.0	N4C2P1	21.0	30	1.40	1.00	0.82	−0.50	2238	2249	2591	−5.0	MA60MA
8/R	54	5.0*∗*2.5	N2C1P1	11.5	9	0.70	0.30	0.30	−1.75	2274	2324	2363	−3.5	MA60MA
8/L	55	3.0*∗*3.0	N3C1P1	17.0	20	0.52	0.22	0.30	−1.50	2419	2342	2465	−3.0	MA60MA
9/L	59	5.0*∗*2.5	N2C1P1	8.0	11	1.30	0.70	0.60	−1.25	3255	3181	2813	−6.0	HumanOpt
10/L	29	3.0*∗*3.0	N3C1P1	13.0	14	0.60	0.15	0.15	−0.25	1660	1559	1647	2.0	HumanOpt
Mean (SD)	46 (14)			11.1 (6.5)	15.8 (9.2)	0.66 (0.31)	0.47 (0.46)	0.60 (0.75)	−1.61 (0.95)	1987 (468)	1905 (524)	1725 (688)	0.8 (4.0)	

m: month(s); BCVA: best corrected visual acuity; Pre: before the triplex surgery; Post: after the triplex surgery; ECD: endothelial cell density; SE: spherical equivalent.

**Table 3:** Characteristics of eyes with severe endothelial cell loss before triplex surgery.

Case/eye	ECD (cells/mm^2^)		Interventions (months after triplex surgery)	Reason analysis			ECD (cells/mm^2^) after DSAEK/PKP				Complications (interval after DSAEK/PKP)
	3 m after triplex surgery	Before DSAEK/PKP		pIOL-related problems	Relative history	Intraop./postop. abnormities of triplex surgery	3 m	6 m	1 y	2 y	
1/R^a^	1579	1234	DSAEK (10)	CE, PO, PAS	PSR		1082	970	835	729	Graft detachment and 1 rebubbling, partial detachment <1/8 remaining

1/L^a^	1045	821	DSAEK (10)	CE, PO, PAS	PSR	Hard nuclear	1348	913	686	605	2 rejection episodes (6 m, 13 m)

2/R^a^	1537	1392	PKP (6)	CE, PO, undersized pIOL, touch of the endothelium and pIOL	TED		1483	1376	1250	1128	

2/L^a^	1172	867	DSAEK (13), PKP (31)	CE, PO, undersized pIOL, touch of the endothelium and pIOL	TED	Endophthalmitis underwent PPV + injection	1035	892	N^c^	1676 (6 m after PKP)	Early rejection, PKP after graft failure. RRD 18 m later and underwent PPV + silicone oil

3/L^a^	996	795	PKP (8)	PO, PAS	PSR	Hyphema intraop.	1377	1245	1013	1034	2 rejection episodes (8 m, 11 m)

5/L	1385	1321^b^	LPC for PCO (16)	Mild chronic inflammation	DM						

10/L	1527	1604^b^	None		PSR						

^a^Eyes that developed corneal decompensation after the triplex surgery.

^b^ECD count one year after triplex surgery.

^c^ECD was unmeasurable.

m: month(s); y: year(s); ECD: endothelial cell density; PKP: penetrating keratoplasty; DSAEK: Descemet's stripping automated endothelial keratoplasty; CE: corneal edema; PO: pupil ovalization; PAS: peripheral anterior synechia; PSR: posterior scleral reinforcement surgery; TED: thyroid eye disease; PPV: pars plana vitrectomy; RRD: rhegmatogenous retinal detachment; LPC: Nd:YAG laser posterior capsulotomy; PCO: posterior capsule opacification; DM: diabetes mellitus.
